# Corrigendum: An accumulation-of-evidence task using visual pulses for mice navigating in virtual reality

**DOI:** 10.3389/fnbeh.2022.1079746

**Published:** 2022-11-22

**Authors:** Lucas Pinto, Sue A. Koay, Ben Engelhard, Alice M. Yoon, Ben Deverett, Stephan Y. Thiberge, Ilana B. Witten, David W. Tank, Carlos D. Brody

**Affiliations:** ^1^Princeton Neuroscience Institute, Princeton University, Princeton, NJ, United States; ^2^Robert Wood Johnson Medical School, New Brunswick, NJ, United States; ^3^Bezos Center for Neural Dynamics, Princeton University, Princeton, NJ, United States; ^4^Department of Psychology, Princeton University, Princeton, NJ, United States; ^5^Department of Molecular Biology, Princeton University, Princeton, NJ, United States; ^6^Howard Hughes Medical Institute, Princeton University, Princeton, NJ, United States

**Keywords:** evidence accumulation, spatial navigation, virtual reality, mouse, behavior, decision making

In the published article, there were typographical errors in one of the equations.

A correction has been made to the second equation in **Materials and Methods**, *Behavioral Task*, Apparatus (page 3). The original equation read:


$$\frac{\text{dθ}}{\text{dt}}=\text{sign }\left(\text{dθ}\right)\times \text{min}([\text{exp}(\text{1}.4\;\times {\text{|dθ|}}^{\text{1}.\text{2}})-1,\pi ])$$

The corrected equation appears below:


$$\frac{\text{dθ}}{\text{dt}}=\text{sign }\left(\text{θ}\right)\times \text{min}([\text{exp}(\text{1}.4\;\times {\text{|θ|}}^{\text{1}.\text{2}})-1],\pi )$$

The authors apologize for this error and state that this does not change the scientific conclusions of the article in any way. The original article has been updated.

## Publisher's note

All claims expressed in this article are solely those of the authors and do not necessarily represent those of their affiliated organizations, or those of the publisher, the editors and the reviewers. Any product that may be evaluated in this article, or claim that may be made by its manufacturer, is not guaranteed or endorsed by the publisher.

