# Supplementary material for: An Accumulation-of-Evidence Task Using Visual Pulses for Mice Navigating in Virtual Reality
Source: Front Behav Neurosci. 2018 Mar 6;12:36. doi: 10.3389/fnbeh.2018.00036 (PMC5845651; doi:10.3389/fnbeh.2018.00036)
Supplement: Supplementary file 2 [file DataSheet1.pdf]

Supplementary Materials

An accumulation-of-evidence task using visual pulses for mice navigating in virtual reality

Lucas Pinto [1,*], Sue Ann Koay [1,*], Ben Engelhard [1], Alice M. Yoon [1], Ben Deverett [1,6], Stephan Y. Thiberge [2], Ilana B. Witten [1,3], David W. Tank [1,2,4,#], Carlos D. Brody [1,4,5,#]

[1] Princeton Neuroscience Institute, [2] Bezos Center for Neural Dynamics, [3] Department of Psychology, [4] Department of Molecular Biology, [5] Howard Hughes Medical Institute, Princeton University, Princeton, NJ, 08544, [6] Robert Wood Johnson Medical School, New Brunswick, NJ

*, # these authors contributed equally to this work

Supplementary Figures, Movie and Table

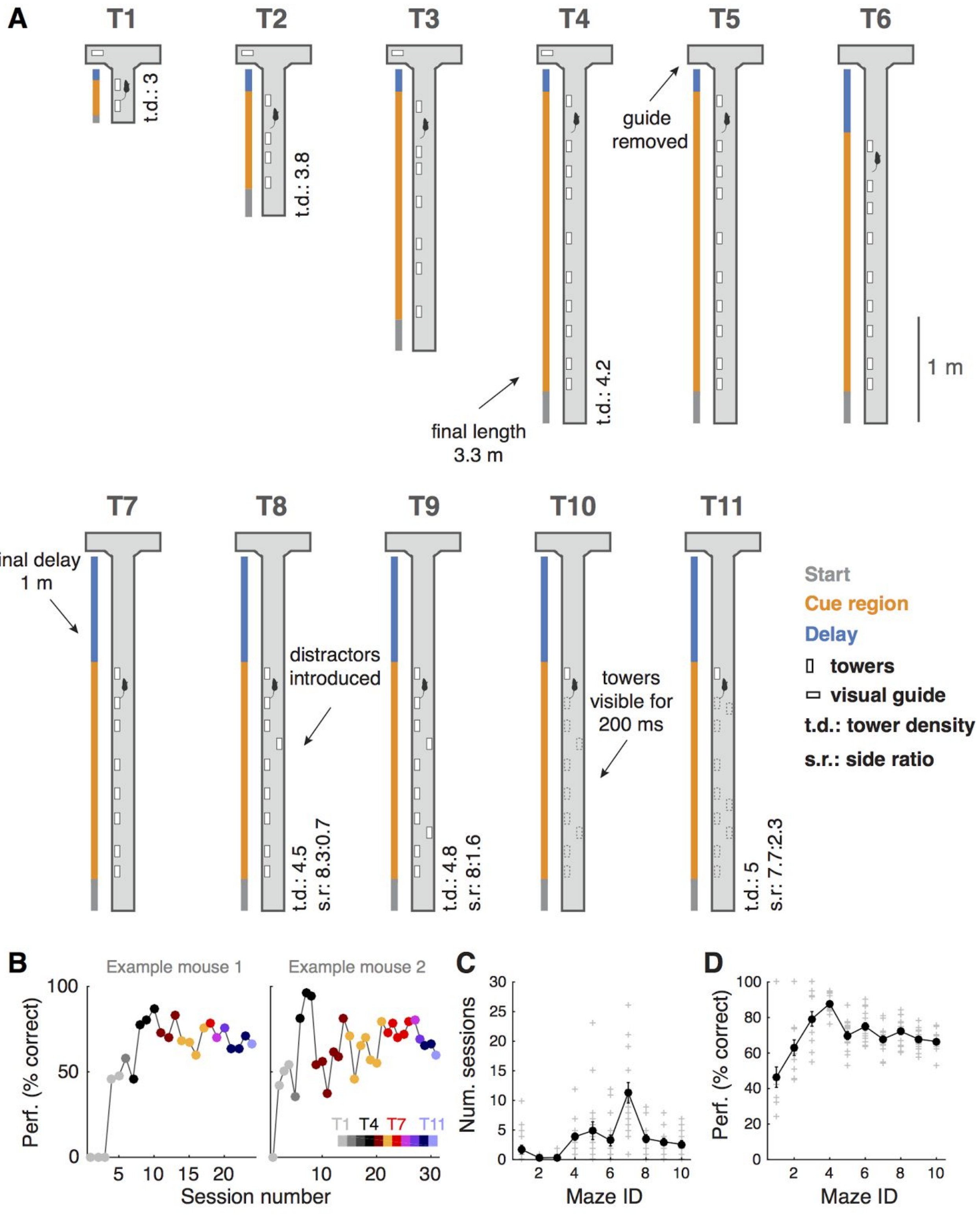

Supplementary Figure 1 | Shaping. (A) Schematic illustration of the 10 different shaping mazes (T1 – T10) and the final accumulation maze (T11). **(B)** Progression through shaping stages of two example mice, where each color indicates a different maze according to the colorbar on the bottom right. **(C)** Number of sessions training sessions spent on each shaping stage. Gray crosses: individual mice, black circles: population mean (n = 17), error bars: ± SEM. **(D)** Average overall performance for each shaping stage. Conventions as in (C).

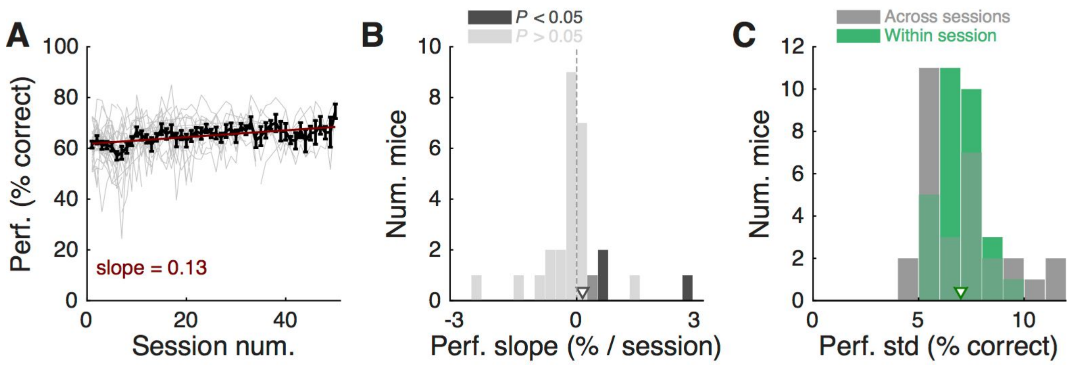

Supplementary Figure 2 | Mice display stable performance over many sessions. (A) Overall performance in the final accumulation maze as a function of session number, for mice with at least 5 sessions (n = 30), and selecting all trials regardless of overall performance (i.e. not applying any performance thresholds). Thin gray lines: individual mice, black line: average across mice, error bars: ± SEM. Red line is best linear fit to average data. **(B)** Distribution of slopes extracted from best-fitting lines to performance of each mouse as a function of session number (i.e. thin gray lines in panel A). Bars are color-coded according to whether the slope is significant (dark gray, i.e. its 95% confidence interval does not overlap zero) or not (light gray). Arrowhead indicates population mean. The distribution was not significantly different from zero (P = 0.99, signed rank test), indicating stable performance across sessions. **(C)** Distribution of standard deviation of average performance across (gray) and within (green) sessions for the mice. Arrowheads: population means. Within-session standard deviation was calculated using performance over a 40-trial running window.

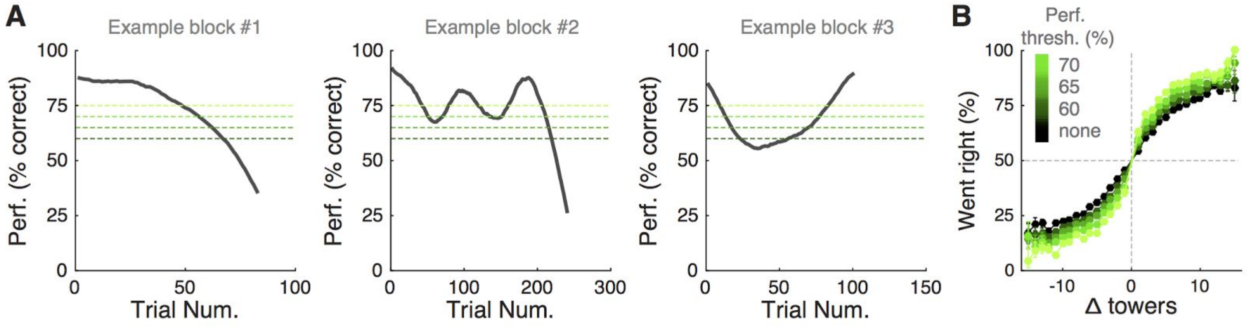

Supplementary Figure 3 | Mice can undergo bouts of high/low performance (A) Three individual examples of consecutive trial blocks on the final accumulation maze, showing performance calculated with a sliding half-Gaussian window (σ = 15 trials), plotted as a function of trial number (trial 1 is the first within the block, not necessarily the first in the session). Dotted lines of different shades of green indicate performance thresholds applied in the analysis shown in D. **(B)** Psychometric curves for aggregate data (metamouse), obtain after excluding trials with performance below different thresholds (illustrated in A).

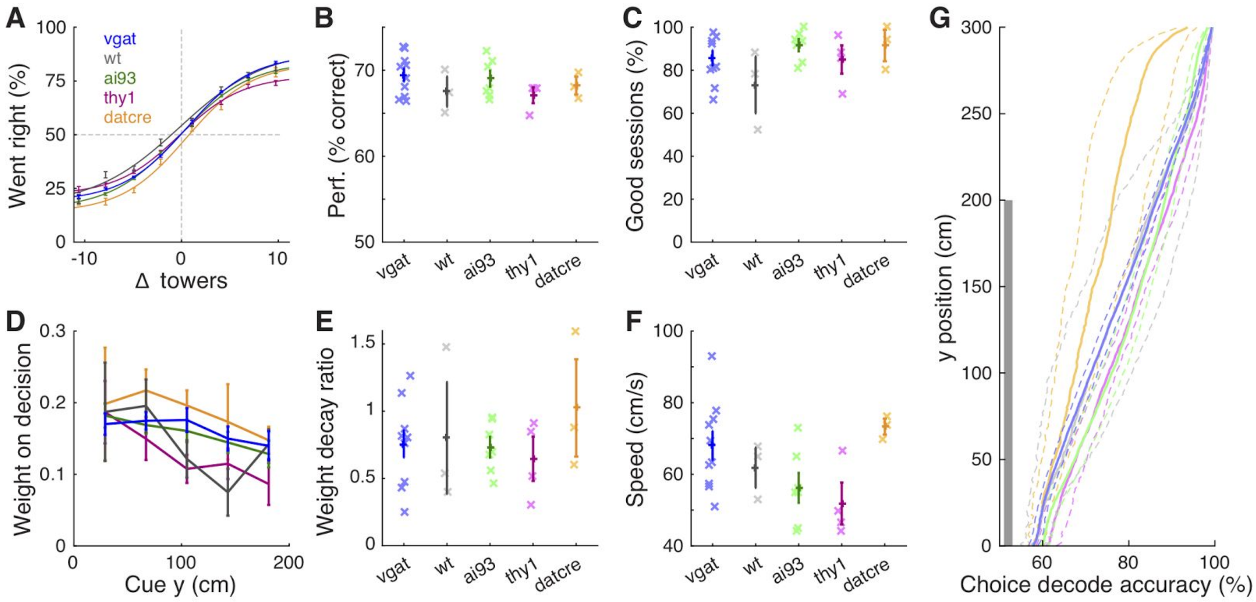

Supplementary Figure 4 | Different mouse strains have comparable performance. (A) Psychometric curves for aggregate data (metamouse) divided into five different strains according to the color code on the top left. Error bars: binomial confidence intervals, lines: best sigmoidal function fits. **(B)** Overall performance averaged across blocks with performance over 60% (see Materials and Methods). Crosses: individual animals, error bars: SEM for each mouse strain. **(C)** Percentage of sessions with at least one trial block over our performance threshold (60%). Conventions as in B. **(D)** Logistic regression of choice on net evidence for each spatial bin. **(E)** Weight decay index according to genotype. Conventions as in B. **(F)** Average running speed, conventions as in B. **(G)** Accuracy of decoding choice from view angle as a function of maze position for different strains. For all but one measure above, there was no significant difference between the different strains, ($P > 0.05$, one-way ANOVA)(Decoding accuracy was measured in the cue period, y < 200 cm). The exception was running speed, significantly different between genotypes (P = 0.04).

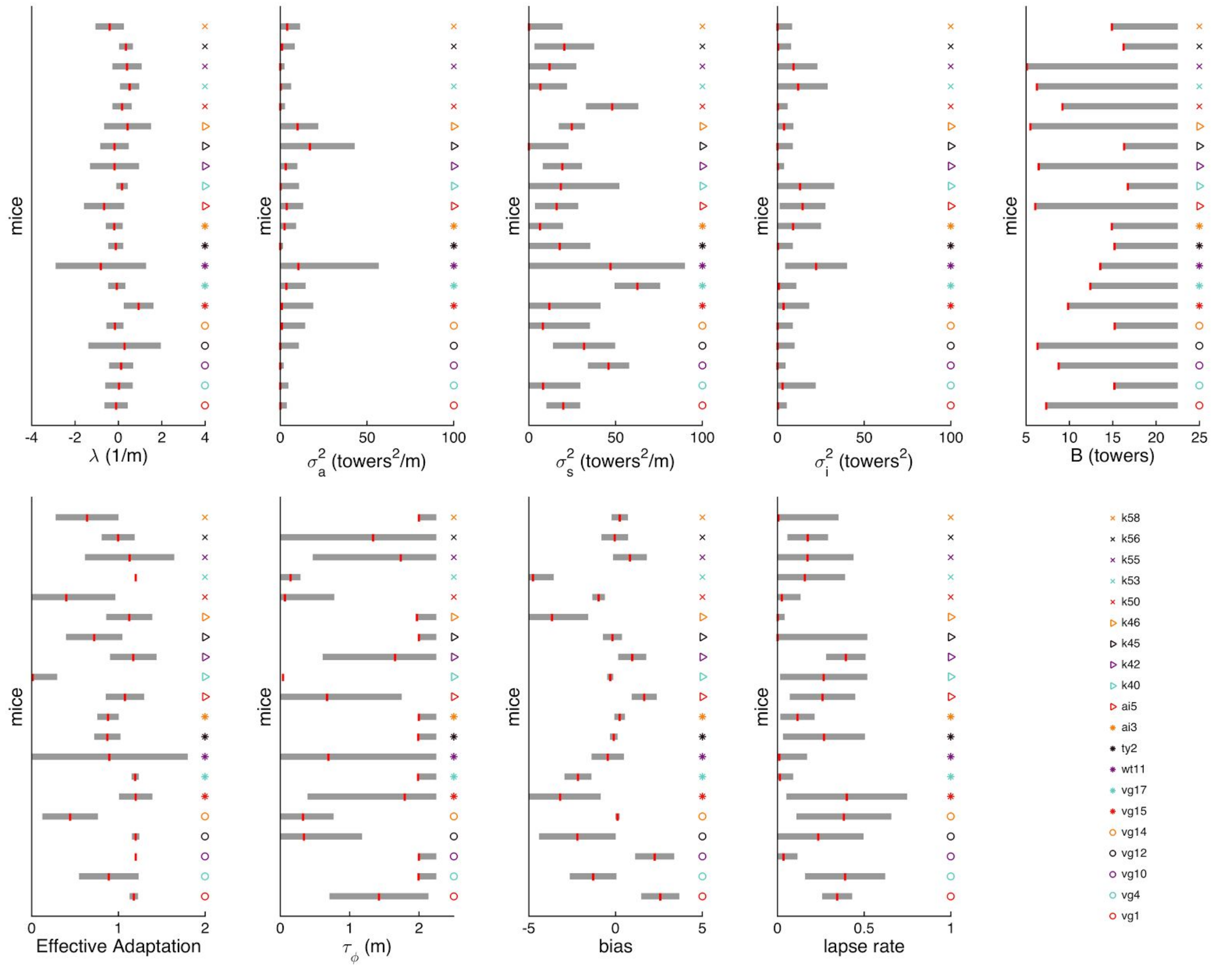

Supplementary Figure 5 | Best-fit parameters for the Brunton et al. model for each mouse. (A) – (I). Vertical red bars indicate the median of best-fit parameters across cross-validation runs, gray shadings indicate one standard deviation of the distribution obtained from cross-validation runs. All panels are sorted according to the same mouse order.

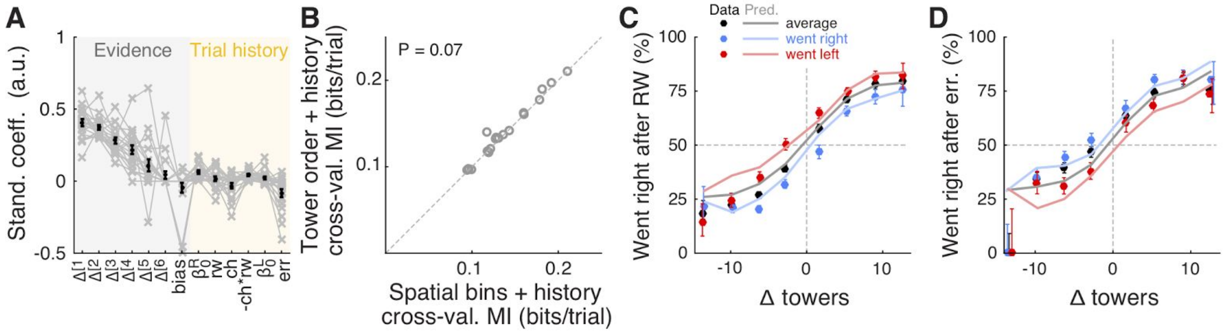

Supplementary Figure 6 | Tower order explains behavior at least as well as position does. (A) Best-fit model coefficients for the tower order model with trial history terms. Thin gray lines: individual mice, thick black lines: population mean, error bars: ± SEM. **(B)** Comparison of cross-validated prediction performance of the spatial bins and tower order models, both with trial history (n = 20 mice). MI: model information index. **(C)** Psychometric curve predictions for an example mouse with large trial history effects, divided according to previous choice in rewarded trials. Circles: data, lines: model prediction. Black: average post-reward curve, blue: trials following rewarded right choices, red: trials following rewarded left choices. Error bars: binomial confidence intervals. **(D)** Psychometric curve predictions for the same mouse in (C), divided according to previous choice in error trials. Conventions as in (C).

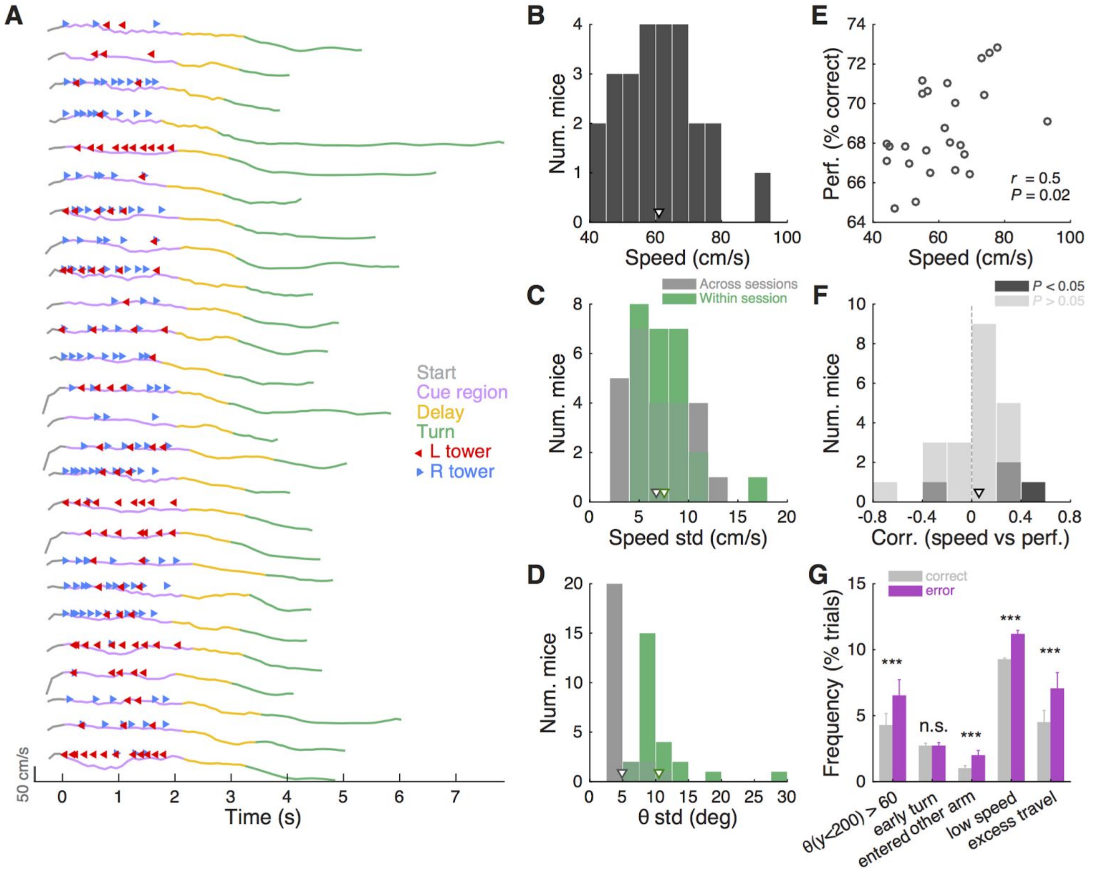

Supplementary Figure 7 | Stability of running patterns. (A) Examples of running speed over time during 25 consecutive trials, aligned by entry in the cue region (t = 0). Each line is color-coded according according to the portion of the maze (start, cue, delay or turn). Tower onset times are shown as leftward red or rightward blue arrows on top of each trace. **(B)** Distribution of average running speed across trials and sessions for animals with at least 1000 trials (n = 25). Arrowhead indicates population mean. **(C)** Distribution of standard deviations of average running speed across session-wide averages (gray, mean ± SEM: 6.7 ± 0.6 cm/s) and across trials within a session (green, mean ± SEM: 7.5 ± 0.6 cm/s). Arrowheads indicate population mean, and follow the same color code. **(D)** Distribution of standard deviations of average view angle across sessions and across trials within a session, calculated separately for right- and left-choice trials and then averaged (mean ± SEM: 4.9 ± 0.4 ˚ vs. 10.4 ± 0.9 ˚, respectively). Conventions as in C. **(E)** Correlation between average running speed and average overall performance across all sessions for each mouse (n = 25, r = 0.48, P = 0.02, Pearson's correlation). **(F)** Distribution of session-wise correlations between average running speed and average overall performance, showing that although there is an overall correlation between the two indicators, for any given mouse there is little correlation of speed and performance on individual sessions: only 4/25 mice had significant correlations between running speed and performance across sessions, and the sign of the correlation was negative for one of these mice (r = 0.06 ± 0.05, mean ± SEM). **(G)** Average frequency of different types of putative motor errors, belonging to five categories: trials with large-magnitude view angles during the cue period (>

60°), trials with early turns (i.e. a turn immediately before the arm, resulting in a wall collision), trials in which the mouse first entered the opposite arm to its final choice, trials with speeds below the 10th percentile (defined separately for each mouse), and trials with traveled distance in excess of 110% of nominal maze length. Frequency was calculated separately for correct and error trials. Error bars, ± SEM. *** $P < 0.001$, n.s.: not significant.

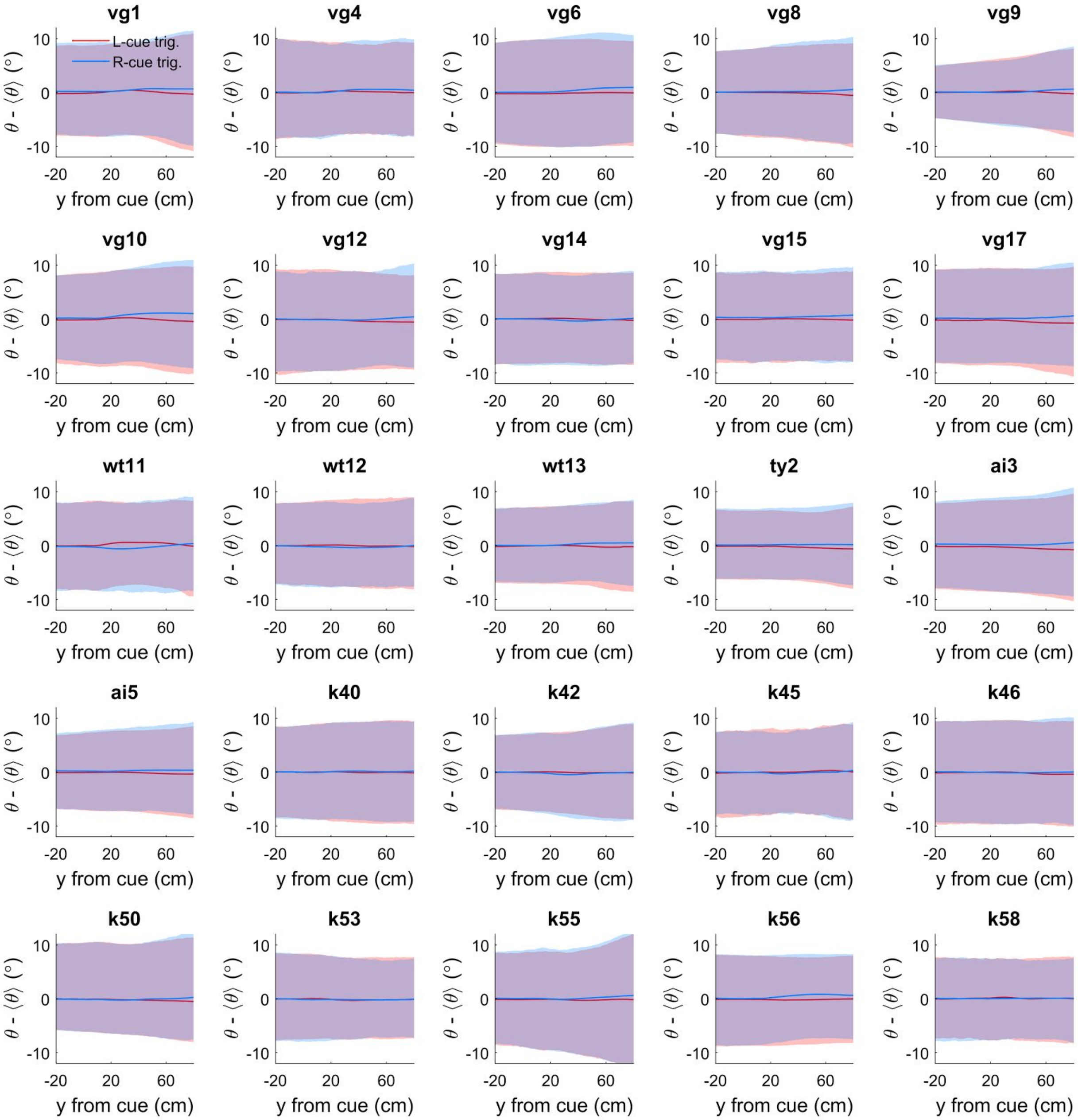

Supplementary Figure 8 | Cue-triggered change in view angles for individual mice. Each panel corresponds to data from a single mouse in this study, otherwise this is the same as Figure 8D: cue-triggered change in the view angle θ relative to the average trajectory ⟨θ⟩ for trials of the same choice. The bands indicate the 1 standard deviation spread across trials, with the lines being the mean across trials.

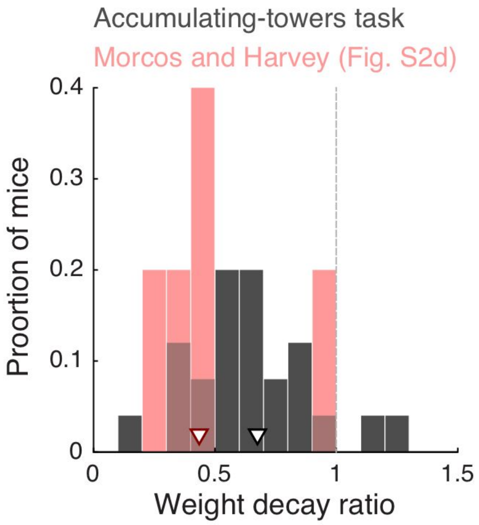

Supplementary Figure 9 | Comparison between the degree of primacy in the accumulating-towers and the Morcos and Harvey tasks. For direct comparison with the Morcos and Harvey task, we recalculated the logistic regression from the final accumulation maze of our task using 6 bins. Data from Supplementary Figure 2, panel d, in Morcos and Harvey (2016) was kindly provided by A.S. Morcos and C.D. Harvey. We then calculated the weight decay ratio as previously described (**Materials and Methods** and **Results, Fig. 3C**). Arrowheads, median.

	T1	T2	T3	T4	T5	T6	T7	T8	T9	T10	T11
**Total length (cm)**	60	170	270	330	330	330	330	330	330	330	330
**Cue period (cm)**	45	120	220	280	280	240	200	200	200	200	200
**Delay (cm)**	10	20	20	20	20	60	100	100	100	100	100
**Tower density (m^{-1})**	3.0	3.8	3.8	3.8	4.2	4.2	4.2	4.5	4.8	4.8	5.0
**Tower duration (ms)**	Inf	Inf	Inf	Inf	Inf	Inf	Inf	Inf	Inf	200	200
**Tower visible from (cm)**	10	10	10	10	10	10	10	10	10	10	10
**Visual guide?**	Y	Y	Y	Y	N	N	N	N	N	N	N
**Tower side ratio (m^{-1})**	Inf	Inf	Inf	Inf	Inf	Inf	Inf	8.3:0.7	8.0:1.6	8.0:1.6	7.7:2.3
**Warm-up**	none	none	none	none	30 T4 trials with < 10% bias and > 80% correct	30 T4 trials with < 10% bias and > 80% correct	30 T4 trials with < 10% bias and > 80% correct	10 T4 and 15 T7 trials with < 10% bias and > 80% correct	10 T4 trials with < 10% bias and > 85% correct	10 T4 trials with < 10% bias and > 85% correct	10 T4 trials with < 10% bias and > 85% correct
**Advancement criteria**	10 completed trials	40 completed trials	80 completed trials at > 60% correct	2 sessions with 100 trials at > 90% correct	1 session with 100 trials at > 80% correct	1 session with 100 trials at > 80% correct	1 session with 100 trials at > 80% correct	1 session with 100 trials at > 75% correct	1 session with 100 trials at > 70% correct	1 session with 100 trials at > 70% correct	n/a
**Easy blocks** (performance calculated over 40-trial window)	none	none	none	none	10 T4 trials if < 70% correct	10 T4 trials if < 70% correct	10 T4 trials if < 70% correct	10 T4 trials if < 65% correct	10 T7 trials if < 60% correct	10 T7 trials if < 60% correct	10 T7 trials if < 55% correct

Supplementary Table 1 | Detailed parameters for all shaping mazes and main accumulation maze.

Supplementary Movie 1. **Playback of six example trials from the accumulating-towers task. Left:** flattened view of the mice's perspective as they navigated the maze. The red lines indicate the estimated boundaries of the binocular field (± 17.5° at the horizon), and the yellow lines indicate ± 45° for reference. θ: view angle. Negative numbers indicate left side by convention. Luminance has been increased for convenience. **Right:** equivalent top-down view of the virtual maze. The mouse avatar turns according to its recorded virtual view angle, and towers become gray outlines when they disappear from the maze. Movie has been slowed down by 2x.

Supplementary Methods

Spatial Poisson distribution of tower locations

We used the following algorithm to randomly generate tower placement locations according to a Poisson process, i.e. with exponentially distributed inter-tower spacings subject to a minimum interval between towers:

Algorithm 1: ***Spatial Poisson process with refractory interval***

Inputs	`L` : Maximum possible location towers<br>`dy` : Minimum possible spacing between towers<br>`mu` : Mean number of towers	
Outputs	`y` : A list of locations of towers. Will be distributed in the range [0,L]	
1	`maxN ← ⌊L/dy⌋`	Draw a random Poisson distributed number that is less than the maximum possible (given the refractory interval)
2	`do`<br>`  n ← random(Poisson(mu))`<br>`until n ≤ maxN`	
3	`Leffective ← L - (n-1)*dy`	Randomly distribute locations within [0,L], but impose a minimum separation
4	`y ← random(Uniform(0,Leffective))`	
5	`y ← sort(y)`	
6	`y ← y * Leffective + (0:n)*dy`	
7	`y ← y + random(Uniform(0,L))`	Randomly rotate to get rid of edge artifacts (non-uniform probability of 0 and L values)
8	`y[y > L] ← y[y > L] - L`	

Exponential gain for translating treadmill movements into changes in virtual view angle

Learning to use a spherical treadmill to execute navigational movements in virtual reality constitutes a substantial portion of the training time for this task. One of the optimizations we have performed to ease this process is to select treadmill-to-virtual-movement transformations so that mice can execute smooth motions without spending aversive amounts of time during turns into the arms of the T-maze. Historically we had first utilized a constant gain (Harvey et al. 2012) for the , but when this gain was low mice required a large amount of time to turn into the arms, encouraging them to initiate turns early (at the expense of accumulating later cues).

Conversely, when this gain was high, small postural shifts in the stem of the T-maze caused the virtual scene to wobble, which was undesirable in a task involving visual cues. These observations motivated the use of a nonlinear gain function that deemphasizes small, uncontrollable movements of the treadmill during running down the stem, but facilitates sharper turns at the end of the T-maze to encourage straighter view angle trajectories.

Heuristic models: optimization technique

Here we defined several models where the choice of the mouse in a series of trials is assumed to be a Bernoulli process parameterized by a probability of making a choice to the right, $p_R = p_R(\vec{x})$, that depends on a set of trial-specific quantities $\vec{x}$ (see **Materials and Methods**).

We obtained best-fit parameters for each model by maximizing the log likelihood of the model for a given dataset comprising of m trials. Let the mouse's choice on the ith trial be $c_i, i = 1, \ldots, m$ which is 1 (0) if the mouse chose right (left), then the likelihood of observing this choice is given by the binomial distribution $B(1, p_R) = p_R(\vec{x}_i)^{c_i}\ [1 - p_R(\vec{x}_i)]^{1-c_i}$. Taking the product of individual-trial likelihoods we obtain:

$$\ln L_x = \sum_{1 \le i \le m} \{c_i\ p_R(\vec{x}_i) \ln p_R(\vec{x}_i) + (1 - c_i) \ln[1 - p_R(\vec{x}_i)]\}$$

Additionally we subtracted L1 penalty terms for all free parameters of the model. For a model that includes all factors, the quantity that is maximized is therefore:

$$\ln L = \ln L_x - \lambda\ (\|\vec{\beta}_\Delta\|_1 + \|\beta_m\|_1 + \|\vec{\beta}_h\|_1)$$

where $\|\vec{y}\|_1 \equiv \sum_i |y_i|$ is the L1 norm. This regularization is used as a method for selecting the most parsimonious model in terms of driving coefficients to zero when they do not result in a significantly better fit for the model (Schmidt, 2010). It was also crucial for some models, particularly those that contain history-dependent lapse terms, because of the presence of multiple local maxima that made the problem otherwise ill-posed.

The regularization strength hyperparameter λ was determined by using a 3-fold cross-validation (CV) procedure to find the optimal model in terms of predictive power. A given dataset was first divided into thirds, and each third is used exactly once as a test set and the remaining two thirds as its complementary training set. To equalize the highly different scales of the $\vec{\Delta}$ factors compared to the rest of the factors which are bounded within [-1,1], for each coordinate the standard deviation $\sigma_i = \sqrt{\langle(\Delta_i - \mu_i)^2\rangle/(m_{2/3} - 1)}$ was computed using the $m_{2/3}$ trials in the training set, and used to scale the evidence factors, $\Delta_i \to \Delta_i/\sigma_i$. In other words, the only thing that this changed was that the coefficients $\vec{\beta}_\Delta$ were expressed in units of $1/\vec{\sigma}$ where $\vec{\sigma}$ are constants derived using the training set (the same are used for the test set, as it would be unfair use of information if they were re-derived for the test set).

Alternative strategy models: one-random-tower analysis details

For the analysis in **Figure 4C**, for each mouse we selected the top 1 third performance blocks, and only analyzed mice that had at last 200 trials in these blocks; we pooled together all trials from these blocks and mice. To test the 1-random tower hypothesis, we reasoned that we expect to obtain a linear psychometric curve when the sum of towers (#R+#L) was fixed for all trials. This is because the probability to go right for the 1-random tower strategy is given by #R/(#R+#L), and if the denominator is fixed, then the psychometric curve (which is given by (#R-#L)/(#R+#L)) is linear in the difference of towers #R-#L, which is the standard x-axis of the psychometric curve. However, we have empirically observed sigmoid shapes for the psychometric curves of the mice's choices. Thus, we proceeded to quantify if the psychometric curves of the mice choices were different from that of the 1-random tower model (as described in **Materials and Methods**). To obtain a dataset with fixed #R+#L, we next selected only trials where #R+#L=12. This number was chosen because it was the maximum number of #R+#L for which there were at least 4000 trials. We then found the psychometric curve for the actual data and the 1-random tower model. As expected, the 1-random tower model results in a linear psychometric curve, whereas the actual data appears more sigmoidal. To find whether these curves are significantly different from each other, we performed a shuffling test in the following way: we generated 5000 bootstrapped pairs of curves by pooling for all trials with a given #R – #L the number of times the mice (or model) chose right, and then randomly assigning the same number of right choices between the two curves, while keeping the total number of trials as in the original data. The sum of absolute differences between the two curves was used as the test statistic.
